# Mobile application: digital health card for deaf adolescents

**DOI:** 10.1590/1980-220X-REEUSP-2023-0366en

**Published:** 2024-09-27

**Authors:** Thiago Yukio Murayama Yasue, Cláudia dos Santos Oliveira, Alessandro Pereira da Silva, Silvia Regina Matos da Silva Boschi, Terigi Augusto Scardovelli, Marcia Aparecida Silva Bissaco, Tabajara de Oliveira Gonzalez, Robson Rodrigues da Silva, Silvia Cristina Martini

**Affiliations:** 1 Universidade de Mogi das Cruzes, Mogi das Cruzes, SP, Brazil.

**Keywords:** Deafness, Adolescent, Mobile Applications, Health Education, Health Records, Personal, Sordera, Adolescente, Aplicaciones Móviles, Educación en Salud, Registros de Salud Personal

## Abstract

**Objective:**

To develop a mobile health application *(mHealth)* accessible to deaf adolescents, based on their health card, promoting autonomy to the access to the user’s health information.

**Method:**

This was a methodological study, divided into three stages: a questionnaire to understand the knowledge of deaf adolescents about the health card, and development of the application using videos in Brazilian Sign Language Libras, Android Studio platform with Java language, and evaluation of the application.

**Results:**

Most deaf adolescents were not aware of the health card. The application has two interface modes: male and female card, with the particularities of each sex. Furthermore, user’s data security is carried out in accordance with the Brazilian General Data Protection Law. The application received a score of 85.5 from experts, being classified as “good” to “excellent” in terms of usability.

**Conclusion:**

The application provides information from the health card in text and video in Libras, according to the selected sex, promoting adolescents’ autonomy in accessing health information. Future implementations may include expansion to other mobile platforms.

## RESUMEN

**Objetivo:**

Desarrollar una aplicación de atención médica móvil *(mSalud)* accesible a adolescentes sordos, a partir de la cartilla de salud del adolescente, promoviendo la autonomía en el acceso a la información de salud del usuario.

**Método:**

Se trató de un estudio metodológico, dividido en tres etapas: cuestionario para comprender los conocimientos de los adolescentes sordos sobre la cartilla de salud, y desarrollo de la aplicación utilizando videos en Libras, plataforma Android Studio con lenguaje Java y evaluación de la aplicación.

**Resultados:**

La mayoría de los adolescentes sordos no conocían su cartilla de salud. La aplicación cuenta con dos modos de interfaz: cartilla masculina y femenina, teniendo las particularidades de cada género. Además, la seguridad de los datos de los usuarios se lleva a cabo de conformidad con la ley general de proteccion de datos. La aplicación recibió una puntuación de 85,5 por parte de los expertos, clasificándola entre “buena” y “excelente” en términos de usabilidad.

**Conclusión:**

La aplicación proporciona información de la cartilla de salud en texto y video en Libras, según el género elegido, promoviendo la autonomía de los adolescentes en el acceso a la información de salud. Las implementaciones futuras pueden incluir la expansión a otras plataformas móviles.

## INTRODUCTION

The World Health Organization (WHO) estimates that by 2050 around 2.5 billion people will be living with some degree of hearing loss, of which at least 700 million will require rehabilitation services^(1)^.

The communication rights of deaf people are protected by law, like those of any citizen. For approximately 466 million people worldwide with hearing loss, the primary form of communication is sign language^(2, 3)^, with Brazilian Sign Language (Libras) being used by deaf Brazilians to facilitate communication. However, due to its distinct grammatical structure in relation to written Portuguese, this communication can sometimes present challenges. One of the strategies would be for the professional to take a LIBRAS course^(4,5,6)^ or have an interpreter at the service, but what happens is that the deaf person goes with a companion, entailing non-compliance with the right to professional secrecy, contradicting the fundamental principles of the Brazilian Public Health System (SUS), which advocates accessibility with equity^(7)^.

The Ministry of Health implements strategies for adolescent health care, including the free distribution of the adolescent health card^(8)^. This card is made available in the SUS Network upon request from the Municipal Health Departments. A specific form has to be filled and sent to the indicated email address^(9)^.

The adolescent health card provides guidance on healthy habits, disease prevention, and specific care, helping adolescents understand their bodies and adopt healthy practices^(10)^. In addition, it provides information on sexuality, family planning, and prevention of sexually transmitted infections. It also addresses early interventions, allowing early identification of health problems and eating disorders, mental disorders, among others. Another important feature is registration, facilitating communication between healthcare professionals and the family.

It addresses another important factor for adolescents, as it helps them to be more responsible with their own health, besides encouraging the search for information and active participation in health-related decisions. This issue poses difficulties to deaf adolescents, as information rarely comes in sign language.

According to studies, adolescents normally do not seek out health services^(11)^, and when they do, they often search for curative care, rather the health promotion and prevention. This is even worse when it comes to deaf patients, as there is a lack of communication between them and the family member, who is normally the one accompanying them during care, or even the healthcare professional themselves^(12)^.

Currently, there are already projects using new technologies for integration, assistance, and humanized care for deaf people in the health area, such as mobile applications and virtual environments for computers. This demonstrates that the use of these tools contributes to the advancement in this area^(13,14,15)^.

The lack of understanding of Brazilian Sign Language (Libras) by many health professionals creates a significant gap in the care provided to deaf adolescents. Even when a deaf adolescent has a cochlear implant, they may still face communication difficulties, just like oral speakers, resulting in a gap between the healthcare professional and the deaf patient. This ineffective communication in healthcare settings can lead to detrimental outcomes such as miscommunication, waste, and errors, as noted in previous studies^(16)^.

During adolescence, a period of intense physical and emotional transformations, young people are in a process of discovery and formation, facing the transition between childhood and adulthood. When searching healthcare facilities, they have to be kindly, respectfully, and ethically treated by health professionals, having their specific needs considered. For deaf adolescents, the communication barrier, often due to a lack of understanding of Libras, can hinder humanized assistance, generating insecurity and resentment during care^(17)^.

Noticing the existence of this gap, the government has been investing in a national policy for people with special needs, such as Law No. 10.436^(18)^, which regulates Libras, Law No. 10.098^(19)^, which certifies access to communication, and the Statute of Children and Adolescents which provides protection for the health of this population, among others.

The hypothesis of the work is that by transforming the information contained in the adolescent health card, using Brazilian Sign Language and video resources, there will be greater interaction between the nurse and the adolescent, allowing greater accessibility to the information.

The literature is still limited on this topic in terms of quality, effectiveness, and suitability of mobile health applications for deaf and disabled people^(20)^.

It is believed that the development of this application, where teenagers can better understand the transformations taking place in their body, sex, and sexuality, without requiring the help of an adult, can contribute to their own knowledge, since sexuality is always a taboo in the family group.

The objective of this paper is to develop a mobile health application *(mHealth)* accessible to deaf adolescents, based on their health card and using videos in Libras, to promote autonomy to the access to the user’s health information.

## METHOD

This is a methodological study focused on developing, validating, and evaluating the application. The study was divided into three stages: a field survey to verify whether there was knowledge about the adolescent health card; the development of the mobile application itself; the evaluation of the application.

### Stage 1 – Knowledge of Deaf Adolescents About the Health Card

The first stage of this project consisted of a quantitative field study that aimed to collect information regarding the knowledge of deaf adolescents about their health card^(8)^. To this end, a demographic questionnaire containing 14 questions was applied to the study participants (adolescents) in the presence of the Nurse Interpreter, an active member of the deaf community, known and trusted by the adolescents involved in the process, and the researcher, to learn about the adolescent’s profile, such as sex, age, aspects of deafness, knowledge about the public health system and the adolescent health card. The study was carried out at the First Baptist Church of Vila Barros in the city of Guarulhos, after approval by the Research Ethics Committee of the Universidade de Mogi das Cruzes.

## SAMPLE DEFINITION

The study population (sample) was made by convenience, consisting of 10 deaf adolescents aged between 10 and 19 years old who participate in a community of the First Baptist Church of Guarulhos Vila Barros. The age range was chosen based on the classification of adolescence of the adolescent health card. Adolescents of both sexes, aged between 10 and 19 years, who had acquired or congenital deafness, with or without knowledge of the adolescent health record, participated in the study. Furthermore, individuals who, despite being deaf, were under 10 years of age or over 19 years of age, and those who did not have total deafness, i.e., who had difficulty hearing or used a hearing aid and/or cochlear implant, were excluded from the study.

The researcher previously scheduled visits to the Baptist Church, where he met those responsible for the adolescents in the classroom. When the adolescents were unable to attend church, meetings were held in their homes between March and April 2021. During these meetings, the objective of the research was explained, focusing on assessing the adolescents’ knowledge about the adolescent health card.

After understanding the purpose of the study, with the authorization of the parents and/or guardians and the adolescent himself through the signing of the consent form, a new date was set, where a questionnaire was distributed with 14 open and closed questions (Chart 1), such as age, sex, reason for deafness and questions about the card. At this time, the parents, the researcher and the nurse and Libras interpreter were present to clarify any doubts.

**Chart 1:** Adolescent demographic questionnaire.

Questions	Possible Alternatives
1 – How old are you?	(Open answer)
2 – What is your sex?	Female, Male
3 – Were you born with deafness, or was it acquired?	(Open answer)
4 – If acquired, what was the reason? If congenital deafness, go to question 5.	(Open answer)
5 – How do you communicate?	Mimicry, Lip Reading, Libras, Writing, Oralized, Drawings, Others
6 – Do you know what the Brazilian Public Health System (SUS) is?	Yes, No
7 – Are you aware of the program that exists in the SUS aimed at adolescent health education?	Yes, No
8 – If yes, which program? If not, skip to question 9.	(Open answer)
9 – Do you know the adolescent’s health card?	Yes, No, I don’t know
10 – If you do, what do you know about the adolescent health card?	Health tips, Healthy eating, Height, My development, Cavities, Clean teeth. Vaccines/Immunization, Puberty, Tanner Stage, Hygiene, Sexuality, Teenage Pregnancy, Safe Sex, Life Project
11 – How did you know the adolescent’s health card?	Nurse, Doctor, Family, Friends, Community, Others
12 – Since what age have you been using the adolescent health card?	(Open answer)
13 – Would you recommend the adolescent’s health card to another adolescent?	Yes, No
14 – If yes, state the main reason.	(Open answer)

## DATA COLLECTION

During the research, the researcher asked questions in Portuguese, while the interpreter/nurse translated into Libras. Five minutes were allowed for closed questions and up to 15 minutes for open questions, considering the need for clear communication for deaf adolescents. When there was uncertainty about knowledge of the booklet, the researcher showed the printed material to clarify. Due to the pandemic, only one adolescent was interviewed at church, while nine were interviewed at home, following safety protocols and adjusting schedules to accommodate families.

### Stage 2: Application Development

The development of the application followed the processes of the waterfall model proposed by Wazlawick^(21)^. For this purpose, the platform *Android Studio* and programming language *Java* were used for the application development. The app is currently in its first version and is featured only for *Android devices,* because that is where most users of smartphones are concentrated in Brazil^(22)^. To record the videos, the tool *VLibras* ^(23)^ was used, where, when inserting text, the software is able to create a 3D animation with an interpreter. The texts and illustrations contained in this application were taken from the teenager’s own card, maintaining its integrity.

### Stage 3: Application Review

After developing the first version of the application, the System Usability Scale (SUS)^(24)^ was applied to IT specialists, as it is a valuable tool for obtaining fast and efficient feedback on the usability of a system, facilitating continuous improvements in design and user experience. This scale assesses the usability of systems by users, measuring their effectiveness, efficiency, and satisfaction when completing tasks. The scale items were formulated in ten affirmative sentences (Table 1, in the “Results” section), following criteria established by NBR ISO 9241-11^(25)^. The response options were formulated based on the Likert scale, allowing five qualitative responses, ranging from “Totally Disagree (0)” to “Totally Agree (4)”, with the intermediate value representing the neutral point.

**Table 1:** Evaluators’ responses according to the System Usability Scale (SUS) – Mogi das Cruzes, SP, Brazil, 2021.

Questions	Strongly agree	Disagree	I neither agree nor disagree	Agree	Strongly agree	
	Reviewers’ response					
1. If I had to, I think I would often use this app.	1	0	1	1	2	5
2. I found the app’s features unnecessarily complex.	2	2	0	0	1	5
3. I think this app is easy to use.	0	0	0	1	4	5
4. I think I would need technical support to be able to use this app frequently.	4	1	0	0	0	5
5. I think the various functions of this app are well integrated with each other.	0	0	1	3	1	5
6. I think there is a lot of inconsistency (navigability, technique) in this app.	2	3	0	0	0	5
7. I think most people could learn how to use this app very quickly.	0	0	0	2	3	5
8. I felt that, initially, I would need the help of a qualified third party to instruct me on how to use the basic functions of the application.	4	1	0	0	0	5
9. I didn’t feel lost when browsing through the app.	0	0	0	0	5	5
10. I would need to learn a lot of things before using this app.	4	0	1	0	0	5

**Figure 1:**
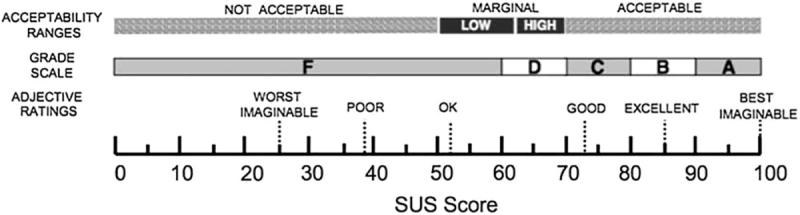
SUS score classification scale.

The score is obtained by considering each item classified from 1 to 5. For odd-numbered items (positive statements), 1 is subtracted from the response value. For even-numbered items (negative statements), the response value is subtracted from 5. The total score is calculated by adding the adjusted values for each item and multiplying them by 2.5, resulting in a total score ranging from 0 to 100 (Figure 1).

## ETHICAL ASPECTS

This work was approved by the Research Ethics Committee of the Universidade de Mogi das Cruzes on November 1, 2020, with opinion 4.374.348, in accordance with Resolution No. 466/2012 of the National Health Council using the Free and Informed Consent Form (FICF).

## RESULTS

### Stage 1: Quantification of Deaf Adolescents’ Knowledge About the Health Card

The time for questionnaire application ranged from 1 to 2.5 hours. Of the 10 participants interviewed, 7 were male and 3 were female, aged between 13 and 18 years (mean and standard deviation of 15.80 ± 2.14), with 100% communication only in Libras.

From the answers provided in the questionnaire, it was observed that 5 (50%) adolescents responded that they were born deaf and the remaining ones, non-deaf. Of those who responded that it was acquired, the reasons were: 1 (10%) had Meningitis at 1 year of age; 2 (20%) were born prematurely and used ototoxic medications (including several medications, such as Gentamicin, an antibiotic widely used in neonatology), which led to deafness; 1 (10%) was diagnosed with pneumonia, needed to use ototoxic medications, and presented deafness as a consequence; and 1 (10%) did not know how to inform the reason.

Regarding knowledge of the Brazilian Public Health System (SUS), 8 (80%) said they knew about it, as they use public services such as hospitals and Primary Health Units (UBS), and in the latter, they only go to the unit to receive vaccinations, and 2 (20%) said they did not know what the SUS is. Of those who knew, 9 (90%) said they were unaware of the adolescent health education program of the Brazilian Public Health System, and therefore did not answer the question (8). Only 1 (10%) reported attending the region’s adolescent care clinic for speech therapy, psychology, and otorhinolaryngology consultations, but was unaware of the program.

Regarding the adolescent health card, 9 (90%) said they had never seen it and 1 (10%) said they had seen it once when a friend showed it to them, but they were unaware of all the topics covered there.

The other questions were not asked, as they were questions that would only be answered if the teenager was familiar with the adolescent health card.

### Stage 2: The Application Developed – Adolescent Health Card

The application “Caderneta do Adolescente” (Adolescent Health Card) is made up of three main screens: home screen, settings screen, and credits screen. Each screen contains interface objects such as: buttons, video player, text boxes, and image boxes. Furthermore, on each screen there is an *ActionBar*, a toolbar located at the top of the layout with easy-to-access buttons.

**Figure 2:**
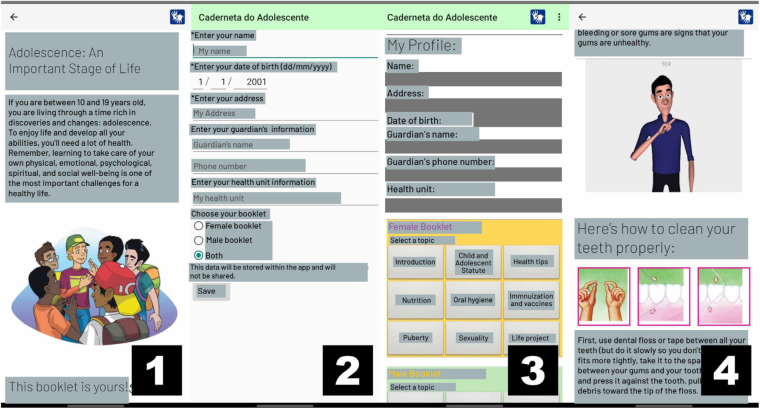
(1) Presentation screen; (2) Settings screen; (3) Home screen; (4) Card topic screen.

The user is directed to a welcome screen Figure 2 (presentation screen) when opening the application for the first time, with a brief explanation about the card and a video with an interpreter in VLibras. There is also information about the accessibility button in Libras. After that, the user accesses the settings screen, where they fill out a profile for the adolescent and choose the type of card they want: female, male or both (Figure 2, settings screen).

After saving the data on the settings screen (Figure 2, settings screen) the user is taken to the application’s home screen, as illustrated in Figure 2 (home screen). All adolescent data entered on the settings screen will be preserved in accordance with the data protection law (Law No. 13.853, of 2019)^(26)^. On this screen, a form with the user’s profile is displayed, containing information such as name, address, date of birth, name and telephone number of the person in charge, and the health unit attended. Below, there is a grid of buttons with the topics of the card (male or female) previously selected in the settings. At the end of the button grid, there is a button to return to the settings screen, where the user can edit or update their profile data if necessary.

On each of the screens (each screen corresponding to a chapter of the card) referring to health topics, information relevant to the adolescent is presented in two different ways, as illustrated in Figure 2 (card topic screen) – through short texts with associated illustrations for easy understanding of the topic and videos in Libras referring to the texts on the screen, which are enabled by clicking on the accessibility button at the top of the page.

### Stage 3: Application Validation

The application validation was carried out digitally by 5 IT experts, where each expert downloaded the application on their mobile device and anonymously answered the usability questionnaire (Table 1).

Through the use of the questionnaire, it was possible to quantify the usability of the application, ending with a score of 85.5 points, out of a possible 100 points, being classified between “good” and “excellent” regarding its usability, according to the SUS scale.

## DISCUSSION

The results of the interview corroborate statements that hearing loss can be present from the moment of birth or develop postpartum or even throughout life, due to some comorbidity^(27)^.

The WHO^(1)^ estimates that 60% of hearing loss problems in children could be avoided with simple measures, such as maternal and neonatal monitoring, screening, and treatment of otitis media, and immunization against rubella and meningitis. In the research, half of the adolescents were born with deafness and half acquired it due to prematurity, use of ototoxic medications or vaccine-preventable diseases. It is important for parents to check their vaccination records from childhood, teaching this habit to their children during adolescence.

Studies corroborate the data found in this research that deafness is one of the most common diseases in the neonatal period^(28)^, affecting 1 to 3 in every 1,000 live births without risk factors, and 20 to 40 in every 1,000 with risk factors. Deafness can be congenital (genetic or acquired) or acquired early during pregnancy, associated with prematurity or exposure to ototoxic substances, such as aminoglycosides, severe perinatal asphyxia, or bacterial meningitis.

In the study, it was found that all interviewees use Brazilian Sign Language as a means of communication, which reinforces the importance of teaching it in undergraduate courses, including health courses.

Despite accessibility policies and laws, there is a glaring gap, especially in adolescent health. Most of those interviewed were unaware of educational programs or the adolescent health booklet, indicating a lack of awareness about these policies. This gap reveals the need for greater dissemination and education about these health resources among deaf adolescents. Only one said he knew about them because a friend in his Biology class had taken it to school and he found it interesting, reporting that “I took a quick look, I don’t know all the content.” This response shows that the deaf adolescents participating in this community are not aware of policies aimed at adolescent health.

The individuality of the deaf adolescent is a relevant aspect, as care often takes place in the presence of a family member, making it difficult to address sensitive topics, such as sexuality, due to the language barrier and the presence of a companion. The use of a digital card in Libras could provide greater comfort and autonomy to adolescents, allowing for more open sharing of personal information. This was also observed in a study where respondents expressed fears and concerns about access to health services and highlighted the need for sign language interpreters^(29)^.

Furthermore, there are still some challenges to be overcome, regarding the acceptance of the adolescent health card by the community, as well as ensuring that it reaches adolescents who do not live in urban areas, or even different socioeconomic groups. Another challenge we are also facing is the resistance from religious entities or even government agencies regarding the content at the end of the card, which shows pictures of sexual organs and even teaches how to use male and female condoms^(30)^.

Briefly speaking, the adolescent health card is an essential tool for promoting health, preventing diseases, and ensuring healthy development during this crucial phase of life, as long as the family and community accept it, as it addresses issues that some families consider to be advanced for their age, for example, if the adolescent is ten years old.

Regarding the validation of the application, it is possible to observe that most of the interviewees agree on the ease of browsing the application screens, in addition to being easy to learn how to use and not requiring prior knowledge for its use. Technology plays a crucial role in implementing and improving the adolescent health card with easy access, as it is digital, where information can be updated more easily or even notifications of possible consultations, vaccinations and exams, among others, can be received. It can have educational videos to explain concepts in a visual and engaging way using sign language. In another work, it was also observed that the majority also preferred videos over text to convey information about their illness and medication, due to their lower levels of learning^(29)^.

It should be highlighted that in this first version, data were stored only on the user’s device, with no internet connection, which contributes to data security in accordance with the General Data Protection Law (Law No. 13.709/2018)^(26)^ which came into force in the country in 2020.

In short, technology can make the card more accessible, educational and engaging, promoting adolescents’ health and well-being, especially for the deaf ones, which was also observed by another study, highlighting that applications *mHealth* of the SUS have significant potential to transform public health in Brazil^(31)^, as they provide several functionalities, such as appointments scheduling, access to test results, information about medicines and vaccination campaigns.

## CONCLUSION

The application (mHealth) “Caderneta do Adolescente” provides information from the health card in text and video in Libras, according to the selected mode, promoting adolescents’ autonomy in accessing health information. The application was classified by experts as “good” or “excellent” regarding its usability, which makes adolescents more independent in accessing information about health, life plans, sexuality, vaccines, among others. Another feature is that the card can be used or consulted by anyone, deaf or not, as well as by teachers and everyone who works with this population or even by hearing adolescents, as the application has texts and images, in addition to videos in Libras. Despite this, until the current status is reflected in a project with all the functions initially proposed developed, it is important to emphasize that future implementations can still be carried out, aiming, for example, at expanding the project to other mobile platforms in addition to the Android operating system.
