# Supplementary material for: Mobile application: digital health card for deaf adolescents
Source: Rev Esc Enferm USP. 2024 Sep 27;58:e20230366. doi: 10.1590/1980-220X-REEUSP-2023-0366en (PMC11433961; doi:10.1590/1980-220X-REEUSP-2023-0366en)
Supplement: Translation

# Translation: Aplicativo móvel: caderneta de saúde digital do adolescente surdo

**DOI:** 10.1590/1980-220X-REEUSP-2023-0366pt

**Authors:** Thiago Yukio Murayama Yasue, Cláudia dos Santos Oliveira, Alessandro Pereira da Silva, Silvia Regina Matos da Silva Boschi, Terigi Augusto Scardovelli, Marcia Aparecida Silva Bissaco, Tabajara de Oliveira Gonzalez, Robson Rodrigues da Silva, Silvia Cristina Martini

**Affiliations:** 1 Universidade de Mogi das Cruzes. Mogi das Cruzes, SP, Brasil.

**Keywords:** Surdez, Adolescente, Aplicativos Móveis, Educação em Saúde, Registros de Saúde Pessoal

## Abstract

**Objetivo:**

Desenvolver um aplicativo móvel de saúde *(mHealth)* acessível a adolescentes surdos, baseado na caderneta de saúde do adolescente, promovendo a autonomia no acesso à informações de saúde do usuário.

**Método:**

Tratou-se de um estudo metodológico, dividido em três etapas: questionário para entender o conhecimento dos adolescentes surdos sobre a caderneta de saúde, e desenvolvimento do aplicativo utilizando vídeos em Libras, plataforma Android Studio com linguagem Java e avaliação do aplicativo.

**Resultados:**

Maioria dos adolescentes surdos não tinha conhecimento da caderneta de saúde. O aplicativo possui dois modos de interface: caderneta masculina e feminina, possuindo as particularidades de cada gênero. Além disso, a segurança de dados do usuário é feita conforme a LGPD. O aplicativo recebeu a nota 85,5 dos especialistas, classificando-o entre “bom” a “excelente” em termos de usabilidade.

**Conclusão:**

O aplicativo fornece informações da caderneta de saúde em texto e vídeo em Libras, de acordo com o gênero escolhido, promovendo a autonomia dos adolescentes no acesso a informações de saúde. Futuras implementações podem incluir a expansão para outras plataformas móveis.

## INTRODUÇÃO

A Organização Mundial da Saúde (OMS) estima que até 2050 cerca de 2,5 bilhões de pessoas viverão com algum grau de perda auditiva, das quais pelo menos 700 milhões necessitarão de serviços de reabilitação^(1)^.

Os direitos de comunicação da pessoa surda são protegidos por lei, como os de qualquer cidadão. Para aproximadamente 466 milhões de pessoas em todo o mundo com perda auditiva, a principal forma de comunicação é a linguagem de sinais^(2,3)^, sendo a Língua Brasileira de Sinais (Libras) utilizada pelos surdos brasileiros para facilitar a comunicação. No entanto, devido à sua estrutura gramatical distinta em relação à língua portuguesa escrita, essa comunicação pode, por vezes, apresentar desafios. Uma das estratégias seria o profissional fazer curso de Libras^(4–6)^ ou ter um intérprete no atendimento; porém, o que acontece é o surdo ir acompanhado, o que acarreta o descumprimento do direito ao sigilo profissional e contradiz os princípios fundamentais do Sistema Único de Saúde (SUS), que preconiza a acessibilidade com equidade^(7)^.

O Ministério da Saúde implementa estratégias de atenção à saúde do adolescente, incluindo a distribuição gratuita da caderneta de saúde do adolescente^(8)^. A disponibilização dessa caderneta na Rede SUS é feita mediante solicitação das Secretarias Municipais de Saúde, com preenchimento de um formulário específico que deve ser enviado para o e-mail indicado^(9)^.

A caderneta de saúde do adolescente oferece orientações sobre hábitos saudáveis, prevenção de doenças e cuidados específicos, auxiliando os adolescentes a compreenderem seus corpos e adotarem práticas saudáveis^(10)^. Além disso, fornece informações sobre sexualidade, planejamento familiar e prevenção de doenças sexualmente transmissíveis. Ela também aborda intervenções precoces, permitindo a identificação precoce de problemas de saúde e distúrbios alimentares, transtornos mentais, entre outros. Outra funcionalidade importante é o registro, facilitando a comunicação entre profissionais de saúde e a família.

Ela aborda outro fator importante para o adolescente, pois aconselha-os a serem mais responsáveis com sua própria saúde, além de incentivar a busca por informações e a participação ativa nas decisões relacionadas à saúde. Este ponto torna-se muito difícil a um adolescente surdo, pois as informações raramente vêm na língua de sinais.

Estudos indicam que adolescentes normalmente não procuram os serviços de Saúde^(11)^, e quando vão, na maioria das vezes procuram de forma curativa, deixando em segundo plano a promoção e prevenção. Isso é ainda piorado quando se trata de paciente surdo, pois existe uma falta de comunicação entre ele e o familiar, que é quem normalmente o acompanha no atendimento, ou mesmo o próprio profissional da saúde^(12)^.

Atualmente, já existem projetos que utilizam novas tecnologias para a integração, auxílio e cuidado humanizado para surdos na área da saúde, como aplicativos móveis e ambientes virtuais para computadores. Isso demonstra que a utilização dessas ferramentas contribui para o avanço dessa área^(13–15)^.

A falta de compreensão da Língua Brasileira de Sinais (Libras) por parte de muitos profissionais de saúde cria uma lacuna significativa no atendimento aos adolescentes surdos. Mesmo quando um adolescente surdo possui implante coclear, ainda pode enfrentar dificuldades de comunicação, assim como os oralizados, resultando em uma lacuna entre o profissional de saúde e o paciente surdo. Essa comunicação ineficaz em ambientes de saúde pode levar a resultados prejudiciais, como falhas na comunicação, desperdício e erros, como observado em estudos anteriores^(16)^.

Durante a adolescência, um período de intensas transformações físicas e emocionais, os jovens estão em processo de descoberta e formação, enfrentando a transição entre a infância e a vida adulta. Ao serem atendidos em unidades de saúde, é essencial que os profissionais os tratem com delicadeza, respeito e ética, considerando suas necessidades específicas. Para adolescentes surdos, a barreira na comunicação, muitas vezes devido à falta de compreensão da Língua Brasileira de Sinais (Libras), pode prejudicar a assistência humanizada, gerando insegurança e ressentimento durante o atendimento^(17)^.

Percebendo essa lacuna, o governo vem investindo em política nacional para pessoas portadoras de necessidades especiais, como por exemplo a Lei n° 10.436^(18)^, que regulamenta Libras, a Lei n° 10.098^(19)^, que certifica o acesso à comunicação, e o Estatuto da Criança e do Adolescente, que confere proteção à saúde dessa população, entre outros.

A hipótese do trabalho é de que ao transformar as informações contidas na caderneta de saúde do adolescente, utilizando a Língua Brasileira de Sinais e recursos de vídeo, existirá uma maior interação entre o enfermeiro e o adolescente possibilitando maior acessibilidade às informações.

A literatura ainda é limitada sobre este assunto em termos de qualidade, eficácia e adequação de aplicativos móveis de saúde para surdos e com deficiência^(20)^.

Acredita-se que o desenvolvimento desse aplicativo onde o adolescente possa entender melhor as transformações que ocorrem no seu corpo, sexo e sexualidade, sem precisar do auxílio de um adulto pode contribuir para seu próprio conhecimento, já que a sexualidade é sempre um tabu no grupo familiar.

O objetivo deste trabalho foi desenvolver um aplicativo móvel de saúde *(mHealth)* acessível a adolescentes surdos, baseado na caderneta de saúde do adolescente e utilizando vídeos em Libras, para promover a autonomia no acesso a informações de saúde do usuário.

## MÉTODO

Trata-se de um estudo metodológico no qual ­preocupou-se em desenvolver, validar e avaliar o aplicativo. O estudo foi dividido em três etapas: uma pesquisa de campo para verificar se existia conhecimento sobre a caderneta de saúde do adolescente; o próprio desenvolvimento do aplicativo móvel; e a a avaliação do aplicativo.

### Etapa 1 – Conhecimento de Adolescentes Surdos Sobre a Caderneta de Saúde

A primeira etapa deste projeto consistiu em um estudo de campo de caráter quantitativo que visou coletar informações referentes ao conhecimento do adolescente com surdez sobre a caderneta de saúde do adolescente^(8)^. Para tanto, aplicou-se um questionário demográfico contendo 14 perguntas aos participantes do estudo (adolescentes) com a presença do Intérprete Enfermeiro, integrante ativo da comunidade surda, conhecido e confiável pelos adolescentes envolvidos no processo, e do pesquisador, para conhecimento do perfil do adolescente, como por exemplo, gênero, idade, aspectos sobre a surdez, conhecimento sobre o sistema único de saúde e da caderneta de saúde do adolescente. O estudo foi realizado na Primeira Igreja Batista de Vila Barros em Guarulhos, após a aprovação do Comitê de Ética em Pesquisa da Universidade de Mogi das Cruzes.

## DEFINIÇÃO DE AMOSTRA

A população do estudo (amostra) foi feita por conveniência, composta por 10 adolescentes com idade entre 10 e 19 anos surdos que participam de uma comunidade da Primeira Igreja Batista de Guarulhos Vila Barros. A idade foi escolhida por ser esta a classificação de adolescente contida na caderneta de saúde do(a) adolescente. Participaram do estudo adolescentes de ambos os gêneros, com idade entre 10 e 19 anos, que tinham surdez adquirida ou congênita, com ou sem conhecimento da caderneta de saúde do adolescente. Além disso, foram excluídos do estudo os indivíduos que, mesmo sendo surdos, tinham idade menor que 10 anos ou maior que 19 anos e aqueles que não apresentavam surdez total, ou seja, ouviam com dificuldade ou com uso do aparelho auditivo e/ou implante coclear.

O pesquisador agendou visitas previamente à Igreja Batista, onde se encontrou com os responsáveis pelos adolescentes na sala de aula. Quando os adolescentes não puderam comparecer à igreja, as reuniões foram realizadas em suas residências entre março e abril de 2021. Durante esses encontros, o objetivo da pesquisa foi explicado, focando na avaliação do conhecimento dos adolescentes sobre a Caderneta da Saúde do Adolescente.

Depois de entender o propósito do estudo, com a autorização dos pais e/ou responsáveis e do próprio adolescente por meio da assinatura do termo de consentimento, foi marcada uma nova data, onde foi distribuído um questionário com 14 perguntas (Quadro 1) abertas e fechadas, como por exemplo, idade, gênero, motivo da surdez e perguntas sobre a caderneta. Neste momento estavam presentes os pais, o pesquisador e o Enfermeiro e intérprete da Libras, para esclarecer eventuais dúvidas.

**Quadro 1:** Questionário demográfico do adolescente.

Perguntas	Alternativas Possíveis
1 – Qual é a sua idade?	(Resposta aberta)
2 – Qual é o seu gênero?	Feminino, Masculino
3 – Você nasceu com surdez, ou foi adquirida?	(Resposta aberta)
4 – Se adquirida, qual foi o motivo? Se surdez congênita, passar para pergunta 5.	(Resposta aberta)
5 – Como você se comunica?	Mímica, Leitura labial, Libras, Escrita, Oralizado, Desenhos, Outros
6 – Você sabe o que é o Sistema Único de Saúde (SUS)?	Sim, Não
7 – Você conhece o programa que existe no SUS voltado para a educação da saúde do adolescente?	Sim, Não
8 – Se sim, qual programa? Se não, pular para pergunta 9.	(Resposta aberta)
9 – Você conhece a caderneta de saúde do adolescente.	Sim, Não, Não sei
10 – Se sim, o que você conhece da caderneta de saúde do adolescente?	Dicas de saúde, Alimentação saudável, Estatura, Meu desenvolvimento, Cárie, Dentes limpos. Vacinas/Imunização, Puberdade, Estágio de Tanner, Higiene, Sexualidade, Gravidez na adolescência, Sexo seguro, Projeto de vida
11 – De que forma você conheceu a caderneta de saúde do adolescente?	Enfermeiro, Médico, Família, Amigos, Comunidade, Outros
12 – Desde que idade você utiliza a caderneta de saúde do adolescente?	(Resposta aberta)
13 – Você indicaria a caderneta de saúde do adolescente para outro adolescente?	Sim, Não
14 – Se sim, cite o principal motivo.	(Resposta aberta)

## COLETA DE DADOS

Durante a pesquisa, o pesquisador fez perguntas em português, enquanto o intérprete/enfermeiro traduzia para Libras. Foram concedidos 5 minutos para perguntas fechadas e até 15 minutos para perguntas abertas, considerando a necessidade de comunicação clara para os adolescentes surdos. Quando havia incerteza sobre o conhecimento da caderneta, o pesquisador mostrava o material impresso para esclarecer. Devido à pandemia, apenas um adolescente foi entrevistado na igreja, enquanto nove foram entrevistados em casa, seguindo protocolos de segurança e ajustando horários para acomodar famílias.

### Etapa 2: Desenvolvimento do Aplicativo

O desenvolvimento do aplicativo seguiu os processos do modelo de cascata proposto por Wazlawick^(21)^. Para tanto, foram utilizadas a plataforma *Android Studio* e a linguagem de programação *Java* para o desenvolvimento do aplicativo. No momento, o aplicativo está na primeira versão, e foi direcionado apenas para dispositivos *Android,* pois é onde se concentra a maioria dos usuários de *smartphones* no Brasil^(22)^. Para a gravação dos vídeos, foi utilizada a ferramenta *VLibras* ^(23)^ onde, ao inserir um texto, o *software* é capaz de elaborar uma animação 3d com um intérprete. Os textos e ilustrações contidos neste aplicativo foram retirados da própria caderneta do adolescente, mantendo sua integridade.

### Etapa 3: Avaliação do Aplicativo

Após o desenvolvimento da primeira versão do aplicativo, a Escala de Usabilidade de Sistema (SUS)^(24)^ foi aplicada a especialistas da área de informática, pois trata-se de uma ferramenta valiosa para obter *feedback* rápido e eficiente sobre a usabilidade de um sistema, facilitando melhorias contínuas no design e na experiência do usuário. Essa escala avalia a usabilidade de sistemas pelos usuários, medindo sua eficácia, eficiência e satisfação ao completar tarefas. Os itens da escala foram formulados em dez frases afirmativas (Tabela 1, na seção “Resultados”), seguindo critérios estabelecidos pela NBR ISO 9241-11^(25)^. As opções de resposta foram formuladas com base na escala Likert, permitindo cinco respostas qualitativas, que variam de “Discordo Totalmente (0)” a “Concordo Totalmente (4)”, com o valor intermediário representando o ponto neutro.

**Tabela 1:** Respostas dos avaliadores de acordo com a Escala de Usabilidade de Sistema (SUS) – Mogi das Cruzes, SP, Brasil, 2021.

Perguntas	Discordo totalmente	Discordo	Não concordo nem discordo	Concordo	Concordo totalmente	
	Resposta dos Avaliadores					
1. Se eu precisasse, eu acho que eu usaria este aplicativo com frequência.	1	0	1	1	2	5
2. Eu achei as funcionalidades do aplicativo desnecessariamente complexas.	2	2	0	0	1	5
3. Eu acho que este aplicativo é fácil de usar.	0	0	0	1	4	5
4. Eu acho que eu precisaria de suporte técnico para poder usar este aplicativo frequentemente.	4	1	0	0	0	5
5. Eu acho que as várias funções deste aplicativo estão bem integradas entre si.	0	0	1	3	1	5
6. Eu acho que há muita inconsistência (navegabilidade, técnica) neste aplicativo.	2	3	0	0	0	5
7. Eu imagino que a maioria das pessoas poderia aprender a usar este aplicativo muito rapidamente.	0	0	0	2	3	5
8. Eu senti que inicialmente, precisaria da ajuda de um terceiro capacitado para me instruir a utilizar as funções básicas do aplicativo.	4	1	0	0	0	5
9. Eu não me senti perdido ao navegar pelo aplicativo.	0	0	0	0	5	5
10. Eu precisaria aprender muitas coisas antes que eu tivesse que sair usando este aplicativo.	4	0	1	0	0	5

A pontuação é obtida considerando-se cada item classificado de 1 a 5. Para os itens ímpares (afirmações positivas), subtrai-se 1 do valor da resposta. Para os itens pares (afirmações negativas), subtrai-se o valor da resposta de 5. O cálculo da pontuação total é feito com a soma dos valores ajustados de cada item e é multiplicada por 2,5, resultando em uma pontuação total que varia de 0 a 100 (Figura 1).

**Figura 1:** Escala de classificação da pontuação SUS.

## ASPECTOS ÉTICOS

Este trabalho foi aprovado pelo Comitê de Ética em Pesquisa da Universidade de Mogi das Cruzes em 01 de novembro de 2020, com o parecer 4.374.348, conforme Resolução n° 466/2012 do Conselho Nacional de Saúde utilizando do Termo de Consentimento Livre e Esclarecido (TCLE).

## RESULTADOS

### Etapa 1: Quantificação do Conhecimento de Adolescentes Surdos Sobre a Caderneta de Saúde

O tempo de aplicação do questionário foi de 1h a 2h30. Dos 10 participantes entrevistados, 7 eram do gênero masculino e 3 do feminino, com idade entre 13 e 18 anos (média e desvio padrão de 15,80 ± 2.14), sendo 100% comunicação apenas em Libras.

A partir das respostas fornecidas no questionário, ­observou-se: 5 (50%) adolescentes, responderam terem nascido surdos e o restante, ouvintes. Aos que responderam ser adquirida, os motivos foram: 1 (10%), teve Meningite com 1 ano de idade; 2 (20%) nasceram prematuros e utilizaram medicamentos ototóxicos (incluem várias medicações, como por exemplo a Gentamicina, um antibiótico muito utilizado em neonatologia) que levaram a surdez; 1 (10%) foi diagnosticado com pneumonia, precisou utilizar medicamentos ototóxicos e apresentou surdez como consequência; e 1 (10%) não soube informar o motivo.

Sobre o conhecimento do Sistema Único de Saúde (SUS), 8 (80%) disseram conhecer, pois utilizam os serviços públicos como hospitais e Unidade Básica de Saúde (UBS), sendo que esta última só é procurada para as vacinações, e 2 (20%) disseram não saber o que é o Sistema Único de Saúde. Dos que conheciam, 9 (90%) disseram desconhecer o programa de educação da saúde do adolescente do Sistema Único de Saúde; por isso, não responderam à questão (8). Apenas 1 (10%) relatou frequentar o ambulatório de assistência do adolescente da região para consulta com fonoaudiologia, psicologia e otorrinolaringologista, mas desconhece o programa.

Com relação à caderneta do adolescente, 9 (90%) disseram que nunca viram e 1 (10%) disse ter visto uma vez quando um amigo mostrou, mas desconhecia todos os assuntos ali abordados.

As demais questões não foram realizadas, por se tratar de perguntas que só seriam respondidas caso o adolescente conhecesse a caderneta do adolescente.

### Etapa 2: O Aplicativo Desenvolvido – Caderneta do Adolescente

O aplicativo “Caderneta do Adolescente” é composto de três telas principais: tela de início, de configurações e de créditos. Cada tela contém objetos de interface, tais como: botões, *player* de vídeo, caixas de texto e caixas de imagens. Além disso, em cada uma existe uma *ActionBar*, uma barra de ferramentas que se localiza no topo do layout com botões de fácil acesso.

O usuário é direcionado para uma tela de boas-vindas, Figura 2 (tela de apresentação), ao abrir o aplicativo pela primeira vez, com uma breve explicação sobre a caderneta e um vídeo com intérprete em VLibras. Há também informação sobre o botão de acessibilidade em Libras. Após isso, o usuário acessa a tela de configurações, onde preenche um perfil do adolescente e escolhe o tipo de caderneta desejada: feminina, masculina ou ambas (Figura 2, tela de configurações).

**Figura 2:** (1) Tela de apresentação; (2) Tela de configurações; (3) Tela Inicial; (4) Tela de tópico da caderneta.

Após salvar os dados na tela de configurações (Figura 2, tela de configurações) o usuário é levado à tela inicial do aplicativo, conforme ilustra a Figura 2 (tela inicial). Todos os dados do adolescente preenchidos na tela de configurações serão preservados de acordo com a lei de proteção de dados (Lei n° 13.853, de 2019)^(26)^. Nesta tela, é exibida uma ficha com o perfil do usuário, contendo informações como nome, endereço, data de nascimento, nome e telefone do responsável, e a unidade de saúde frequentada. Abaixo, há uma grade de botões com os tópicos da caderneta (masculina ou feminina) selecionada anteriormente nas configurações. No final da grade de botões, há um botão para retornar à tela de configurações, onde o usuário pode editar ou atualizar seus dados de perfil, se necessário.

Em cada uma das telas (sendo que cada tela corresponde a um capítulo da caderneta) referentes aos tópicos de saúde, as informações pertinentes ao adolescente são apresentadas de duas maneiras distintas, como ilustra a Figura 2 (tela de tópico da caderneta) – por meio de textos curtos com associação de ilustrações para fácil compreensão do tema e vídeos em Libras referentes aos textos da tela, que são habilitados ao clicar no botão de acessibilidade no topo da página.

### Etapa 3: Validação do Aplicativo

A validação do aplicativo foi realizada digitalmente por 5 especialistas da área da informática, onde cada especialista baixou o aplicativo em seu dispositivo móvel e respondeu anonimamente ao questionário de usabilidade (Tabela 1).

Por meio do uso do questionário, foi possível quantificar a usabilidade do aplicativo, finalizando com a pontuação de 85,5 pontos, de 100 pontos possíveis, sendo classificado entre “bom” e “excelente” quanto a sua usabilidade, conforme escala SUS.

## DISCUSSÃO

Os resultados da entrevista corroboram as afirmações de que a deficiência auditiva pode estar presente desde o momento do parto ou desenvolver no pós-parto ou mesmo ao longo da vida, devido a alguma comorbidade^(27)^.

A OMS^(1)^ estima que 60% dos problemas de perda auditiva em crianças poderiam ser evitados com medidas simples, como acompanhamento materno e neonatal, triagem e tratamento de otite média, e imunização contra rubéola e meningite. Na pesquisa, metade dos adolescentes nasceu com surdez e metade adquiriu devido a prematuridade, uso de medicações ototóxicas ou doenças imunopreveníveis. Destaca-se a importância de os pais consultarem a carteira de vacinação desde a infância, ensinando esse hábito aos filhos para a adolescência.

Estudos corroboram os dados encontrados nesta pesquisa de que a surdez é uma das doenças mais comuns no período neonatal^(28)^, acometendo de 1 a 3 em cada 1.000 nascidos vivos sem fatores de risco, e 20 a 40 em cada 1.000 com fatores de risco. A surdez pode ser congênita (genética ou adquirida) ou precocemente adquirida durante a gestação, associada à prematuridade ou exposição a substâncias ototóxicas, como aminoglicosídeos, asfixia perinatal grave ou meningite bacteriana.

No estudo, constatou-se que todos os entrevistados utilizam a Língua Brasileira de Sinais como meio de comunicação, o que reforça a importância do seu ensino nas graduações, incluindo os cursos de saúde.

Apesar das políticas e leis de acessibilidade, há uma falha evidente, especialmente na saúde dos adolescentes. A maioria dos entrevistados desconhece programas educacionais ou a caderneta de saúde do adolescente, indicando uma falta de conscientização sobre essas políticas. Essa lacuna revela a necessidade de maior divulgação e educação sobre esses recursos de saúde entre os adolescentes surdos. Apenas um disse que conhecia porque um amigo na aula de biologia havia levado para a escola, e havia achado interessante, e relata “dei uma olhada rapidamente, não conheço todo o conteúdo”. Essa resposta mostra que os adolescentes surdos participantes desta comunidade não têm conhecimento das políticas voltadas para a saúde do adolescente.

A individualidade do adolescente surdo é um aspecto relevante, pois muitas vezes o atendimento ocorre na presença de um familiar, dificultando a abordagem de temas delicados, como sexualidade, devido à barreira linguística e à presença do acompanhante. A utilização da caderneta digital em Libras poderia proporcionar maior conforto e autonomia aos adolescentes, permitindo um compartilhamento mais aberto de informações pessoais. Isso também foi observado em um estudo onde entrevistados expressaram receios e preocupações sobre o acesso aos serviços de saúde e salientaram a necessidade de intérpretes de linguagem gestual*(29)*.

Além disso, existem alguns desafios a serem ainda percorridos, quanto à aceitação da caderneta de saúde do adolescente pela comunidade, como também fazer com que ela chegue até o adolescente que não mora em áreas urbanas, ou mesmo aos pertencentes a diferentes grupos socioeconômicos. Outro desafio também encontrado é a resistência de entidades religiosas ou mesmo de órgãos governamentais quanto ao conteúdo do final da caderneta, onde aparecem figuras dos órgãos sexuais e ou mesmo o ensinamento do uso de camisinhas feminina e masculina^(30)^.

Em resumo, a caderneta de saúde do adolescente é uma ferramenta essencial para promover a saúde, prevenir doenças e garantir um desenvolvimento saudável durante essa fase crucial da vida, desde que a família e a comunidade a aceite, pois ela aborda assuntos que algumas famílias acham avançados para a idade, como por exemplo se o adolescente tiver dez anos.

A respeito da validação do aplicativo, é possível observar que a maior parte dos entrevistados concorda quanto à facilidade de se navegar nas telas do aplicativo, além de ser fácil de aprender a utilizar e não necessitar de conhecimento prévio para seu uso. A tecnologia desempenha um papel crucial na implementação e aprimoramento da caderneta de saúde do adolescente, com a facilidade do acesso, por ser digital, onde as informações podem ser atualizadas com mais facilidade ou mesmo por poder receber notificações de possíveis consultas, vacinas e exames, entre outros. Ela pode ter vídeos educativos para explicar conceitos de forma visual e envolvente utilizando a linguagem de sinais. Em outro trabalho também se observou que a maioria preferiu vídeos em vez de texto para transmitir informações sobre a sua doença e medicação, devido aos seus níveis mais baixos de aprendizado^(29)^.

É importante destacar que, nesta primeira versão, os dados ficam armazenados apenas no dispositivo do usuário, não havendo conexão com a internet, o que contribui para a segurança dos dados de acordo com a Lei Geral de Proteção de Dados (Lei n° 13.709/2018)^(26)^ que entrou em vigor no país em 2020.

Em resumo, a tecnologia pode tornar a caderneta mais acessível, educativa e envolvente, promovendo a saúde e o bem-estar dos adolescentes, principalmente o surdo, o que foi observado também em outro estudo, que destacam que os aplicativos *mHealth* do SUS têm potencial significativo para transformar a saúde pública no Brasil^(31)^, pois oferecem várias funcionalidades, como agendamento de consultas, acesso a resultados de exames, informações sobre medicamentos e campanhas de vacinação.

## CONCLUSÃO

O aplicativo (*mHealth*) “Caderneta do Adolescente” fornece informações da caderneta de saúde em texto e vídeo em Libras, de acordo com o gênero escolhido, promovendo a autonomia dos adolescentes no acesso a informações de saúde. O aplicativo foi classificado pelos especialistas entre “bom” e “excelente” em relação a sua usabilidade, o que torna o adolescente mais independente ao acesso a informações de saúde, projeto de vida, sexualidade, vacinas, entre outros. Outra funcionalidade é que a caderneta poderá ser utilizada ou consultada por qualquer pessoa, surdos ou não, além de professores e todos que trabalham com essa população, ou até mesmo pelo adolescente ouvinte, pois o aplicativo possui textos e imagens, além dos vídeos em Libras. Apesar disso, até o estado atual refletir em um projeto com todas as funções inicialmente propostas elaboradas, é importante ressaltar que futuras implementações ainda podem ser efetuadas, visando, por exemplo, a expansão do projeto para outras plataformas móveis além do sistema operacional Android.
